# Intercellular crosstalk mediated by tunneling nanotubes between central nervous system cells. What we need to advance

**DOI:** 10.3389/fphys.2023.1214210

**Published:** 2023-08-21

**Authors:** D. L. Capobianco, L. Simone, M. Svelto, F. Pisani

**Affiliations:** ^1^ Department of Biosciences, Biotechnologies and Environment, University of Bari “Aldo Moro”, Bari, Italy; ^2^ Fondazione Istituto di Ricovero e Cura a Carattere Scientifico (IRCCS) Casa Sollievo della Sofferenza, Cancer Stem Cells Unit, San Giovanni Rotondo, Italy; ^3^ Center for Synaptic Neuroscience and Technology, Istituto Italiano di Tecnologia, Genova, Italy

**Keywords:** intercellular communication, tunneling nanotubes, central nervous system, *in-vitro* 3D model, super resolution live-cell microscopy

## Abstract

Long-range intercellular communication between Central Nervous System (CNS) cells is an essential process for preserving CNS homeostasis. Paracrine signaling, extracellular vesicles, neurotransmitters and synapses are well-known mechanisms involved. A new form of intercellular crosstalk mechanism based on Tunneling Nanotubes (TNTs), suggests a new way to understand how neural cells interact with each other in controlling CNS functions. TNTs are long intercellular bridges that allow the intercellular transfer of cargoes and signals from one cell to another contributing to the control of tissue functionality. CNS cells communicate with each other via TNTs, through which ions, organelles and other signals are exchanged. Unfortunately, almost all these results were obtained through 2D *in-vitro* models, and fundamental mechanisms underlying TNTs-formation still remain elusive. Consequently, many questions remain open, and TNTs role in CNS remains largely unknown. In this review, we briefly discuss the state of the art regarding TNTs identification and function. We highlight the gaps in the knowledge of TNTs and discuss what is needed to accelerate TNTs-research in CNS-physiology. To this end, it is necessary to: 1) Develop an *ad-hoc* TNTs-imaging and software-assisted processing tool to improve TNTs-identification and quantification, 2) Identify specific molecular pathways involved into TNTs-formation, 3) Use *in-vitro* 3D-CNS and animal models to investigate TNTs-role in a more physiological context pushing the limit of live-microscopy techniques. Although there are still many steps to be taken, we believe that the study of TNTs is a new and fascinating frontier that could significantly contribute to deciphering CNS physiology.

## 1 Introduction

Cell-to-cell communication is essential for preserving tissue functions and homeostasis. A variety of mechanisms are involved: nearby cells can communicate with each other via connexin-formed gap junctions (GJs) and synapses, while distant cells can also do so via paracrine secreted signals and extracellular vesicles.

In 2004, Rustom and colleagues discovered a new form of long-range cell-to-cell crosstalk mechanism based on long plasma membrane bridges named tunneling nanotubes (TNTs) (Rustom et al., 2004). *In vitro*, TNTs have been described as non-adherent, actin-based cytoplasmic extensions that serve as long-distance membranous bridges. Rustom and others have demonstrated the presence of TNTs in various cell types, indicating that TNTs are a widespread cellular phenomenon crucial for long-distance cell-to-cell communication. TNTs allow the intercellular transfer not only of small molecules, such as ions, second messengers, and metabolic substrates, but also of macromolecules, including proteins and nucleic acids, and organelles. This results in a cell “support” network that plays a role in the control of tissue functions (reviewed in (2)). For instance, mounting evidence suggests that TNT-mediated intercellular mitochondrial transfer can protect recipient cells from bioenergetic deficit and apoptosis, which can be caused by pathological factors (Pisani et al., 2022).

TNTs have been observed in various cell types of the Central Nervous System (CNS) (Khattar et al., 2022), including neurons, glial cells, pericytes, and brain endothelial cells as listed in Table 1. Data suggest that TNTs could play important roles in the maintenance of neuronal networks. In this regard it has been demonstrated that TNTs contribute to the transmission of electrical signals, to the regulation of immune responses in the CNS and could play a role during brain development (Wang et al., 2010; Wang et al., 2012; Wang and Gerdes, 2012; Zurzolo, 2021; Cordero Cervantes et al., 2023). Furthermore, we have recently shown that TNTs-based crosstalk occurs between human blood-brain barrier cells (Pisani et al., 2022). In pathological conditions affecting the CNS, such as Alzheimer’s and Parkinson’s diseases, TNTs may also play a role in the spread of pathological proteins (Abounit et al., 2016; Tardivel et al., 2016; Rostami et al., 2017; Dilna et al., 2021).

**TABLE 1:** TNTs, in cell types of Central Nervous System.

CNS cell type	Model	Conditions	Cargoes	TNTs connected cells	TNTs identification and quantification	Software-assisted TNTs quantification	Mechanism of TNTs formation	3D-CNS or animal models	References
Brain endothelial cells	Murine brain sections	Hypoxia-Ischemia	None	Endothelial-endothelial	Confocal microscopy	No	No	Yes	Girolamo et al. (2023)
Neurons,	*In vitro* 2D model	Parkinson disease	α-Synuclein (α-Syn)	Neuron-microglia	Confocal microscopy	No	No	No	Chakraborty et al. (2023b)
Microglia cells									
Astrocytes,	*In vitro* 2D model	Physiological and ischemic stroke	mitochondria	Astrocyte-pericyte. Endothelial cells-pericytes.	Confocal microscopy	No	No	No	Pisani et al. (2022)
Brain Pericytes,									
Brain Endothelial cells.									
Astrocytes	Murine models	Physiological	EGFP	Astrocytes-neurons	Immunoelectron microscopy and fluorescence microscopy.	No	No	No	Chen and Cao (2021)
Pericytes	Human	Physiological and pathological angiogenesis	None	Pericyte-endothelial cells	Confocal microscopy	No	No	No	Errede et al. (2018)
	Brain								
	sections								
Pericytes	Murine models	Neurovascular coupling	Ca^2+^	Pericyte- pericyte	Live-animal two-photon microscopy	No	No	Yes	Alarcon-Martinez et al. (2020)
Microglia	*In vitro* 2D model, murine model.	Parkinson disease	α-Synuclein (α-Syn)	Microglia-microglia	Flow cytometric analysis	No	No	Yes	Scheiblich et al. (2021)
					*In vivo* two-photon imaging				
					Fluorescence microscopy				
Astrocytes	*In vitro* 2D model	Physiological	EGFP	Astrocytes-astrocytes	Live-cell confocal microscopy	No	Yes	No	Sun et al. (2012)
Neuron	*In vitro* 2D model	Physiological	None	Neuron-astrocytes	Differential interference contrast microscopy.	No	No	No	Wang et al. (2012)
Astrocytes					Fluorescent microscopy.				

Despite the many data published on this new form of intercellular communication mechanism between neural cells, the role of TNTs in the CNS remains largely unclear. Further investigation and new tools are required to accelerate our understanding of TNTs’ role in the CNS.

**FIGURE 1:**
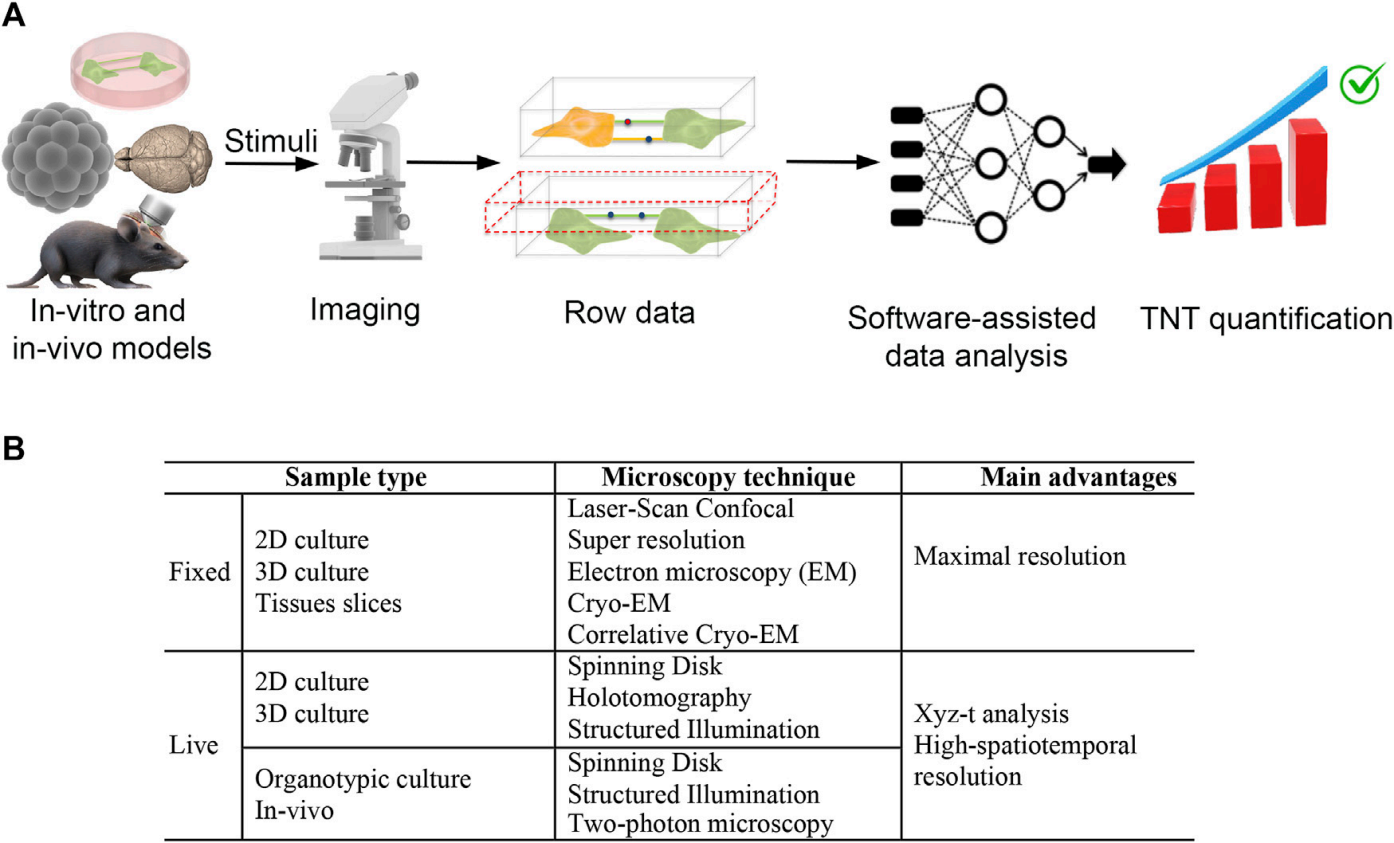
Ideal workflow and imaging techniques to deciphering tunneling nanotubes mediated crosstalk between central nervous system cells. **(A)** To make progress in understanding the triggering factors and functions of TNTs in the central nervous system, we require an integrated system including: 1) specific *in vitro* and *in vivo* models, 2) super-resolution live microscopy and, 3) software-assisted data analysis. *In vitro* and *in vivo* models are valuable for investigating the intercellular transfer of cargoes such as ions, RNAs, proteins, and organelles. High-resolution live-cell confocal microscopy techniques, including spinning disk and structured illumination super-resolution microscopy, are used to analyze these models. The data produced by 3D reconstruction are then analyzed using artificial intelligence (AI) approaches to obtain unbiased TNT quantification. For *in vitro* data, the AI-based software must be capable of discriminating TNT as F-actin positive, straight structures detached from the substrate that are capable of transferring cargo from one cell to another. With the use of these tools, we can quickly and objectively test candidate stimuli that trigger or destroy TNTs. Ultimately, this approach could speed up the identification of specific molecular pathways involved in TNT formation and the functions of TNTs in the CNS. **(B)** Sample type and imaging techniques for efficient TNTs analysis in fixed and live samples.

In this review, we briefly discuss the state of the art regarding the identification and function of TNTs. We highlight the gaps in our knowledge of TNTs and, more importantly, what is needed, in our opinion, to accelerate the study of TNT-mediated intercellular crosstalk in the CNS (Figure 1).

## 2 Discussion

### 2.1 TNTs-identification and quantification

#### 2.1.1 We need a rapid, automatic and operator-independent method

TNTs are described as (I) thin (20–700 nm) and straight membranous protrusions hovering over the substrate and directly connecting two (or more) cells of either the same (homotypic) or different (heterotypic) types. They (II) contain F-actin cytoskeletal filaments and (III) are able to transfer cargo from one cell to another (Cordero Cervantes and Zurzolo, 2021).

Although some authors have reported the identification of potential TNTs markers (Jung et al., 2017), no specific markers exclusive to TNTs have been discovered to date. For this reason, morpho-functional analysis continues to be the primary standard for TNT identification and quantification.

Currently, high-resolution confocal microscopy followed by 3D-reconstruction and Z-stacks analysis, live-cell fluorescence microscopy, and analysis of intercellular transfer of cellular cargoes are the main approaches used to identify and quantify TNTs (as detailed in Table 1). These approaches are time-consuming and dependent on the researcher’s expertise and interpretation. Furthermore, fixed-cell or fixed-tissue based approaches are affected by fixation procedures that tend to break TNTs.

An automatic or semi-automatic TNT identification and quantification method is mandatory to boost TNT studies. To this aim, a method aimed at developing automated detection of TNTs using Z-stacks confocal images was reported in a paper published in 2006 (Hodneland et al., 2006). More recently, a machine learning approach potentially useful for TNT quantification was developed by Smirnov and colleagues to study the dynamics of dendritic spines using live-cell microscopy data (Smirnov et al., 2018). A deep-learning artificial intelligence (AI) approach was first proposed by Ceran and colleagues for TNT analysis (Ceran et al., 2022). All these methods were able to identify only 50%–60% of human expert-identified TNTs.

For these reasons, to date, manual TNT counting based on trained investigators based on fixed and live-cell microscopy analysis remains the gold-standard method. This strongly slows the study of TNTs.

Furthermore, only phase-contrast images or plasma membrane fluorescent dyes of single-cell type culture, were used as input data in these methods. No F-actin staining, cargo tracking analysis, or intercellular transfer of cellular cargoes were investigated. Since these are three TNT-related properties that distinguish TNTs from other cell protrusions, whether these approaches are really able to automatically quantify TNTs remains elusive. We believe that taking these additional elements into account could contribute to significantly increase and improve the automatic quantification of TNTs. To achieve this objective, cell culture experiments should be conducted using co-cultures of donor and receiving cells, and the analysis should be performed using live-cell time-lapse confocal microscopy in 4D mode (xyz-t). Tracking analysis, utilizing dedicated software like TrackMate, a plugin of the free software Fiji, can assist in describing the trajectory of cargo in the xyz dimensions over time. The main challenge lies in implementing these analyses within the software design of AI-based approaches. We hope in fact that recent progress in AI-based methods will help us in this hard work. This point is crucial to accelerate the identification of molecules and stimuli that can affect or enhance the formation of TNTs, accelerating, for instance, the decoding of molecular pathways involved in TNT formation and the identification of TNT functions.

## 3 How cells generate TNTs is unclear

### 3.1 We need more molecular insights to design more TNTs-specific experiments

The formation of TNTs is thought to occur via two main mechanisms: 1) cell dislodgement, in which two cells that were initially attached to one another separate, leaving a membrane thread that develops into an actin-supported TNT, and 2) actin-driven, in which a cell forms a precursor protrusion through actin assembly that subsequently fuses with a recipient cell, forming a TNT. However, it is possible that there are other mechanisms involved in the formation of TNTs that have yet to be discovered. There are still many questions that remain unanswered in order to fully understand this process. For example, what are the actin regulators orchestrating the formation of TNTs? Do TNTs mature from a filopodia-like precursor, or is the actin-driven mechanism of TNT formation a *de novo* process? Nina Ljubojevic et al. in Ljubojevic et al. (2021).

According to imaging studies on cultured cells, TNTs can initially form from thin, actin-driven protrusions that resemble fingers (filopodia) or from a direct contact between cell bodies. The resulting TNTs maintain an intercellular distance that can be up to five hundred times greater than the TNT’s thickness (hundreds of nanometers) while suspended between the cells.

A highly contentious issue is whether a TNT is open-ended or close-ended *in vivo* as well as how short and dynamic filopodia transform into long and stable TNTs, and what causes the filopodia to change into TNTs. One of the newest hypotheses for the formation of TNTs, especially the close-ended type, was recently reported by Minhyeok Chan and colleagues. In this paper, the authors found that TNTs develop from a double filopodial intercellular bridge (DFB) between distant cells. The author shows that DFB results from the dimerization of N-cadherin extracellular domains from two different filopodia of distant cells and that this bridge evolves into a closed-ended TNT. The transition from a DFB to a close-ended TNT appears to be triggered by mechanical energy accumulated in a twisted DFB. Myosin V and/or myosin II seem to play a functional role in this twisting mechanism. A specific biophysical mechanism of action was proposed, and the elastic properties of TNTs and DFB were experimentally measured (Chang et al., 2022). This intriguing paper highlights the synergistic role of F-actin/N-cadherin and myosin in TNT formation. Most recently, was found that F-actin and cadherin connection control the tensile strength and flexural strength of TNTs, respectively (Li et al., 2022). It is important to note that, before these papers, the presence of N-cadherin and Myosin 10 in TNT structures was found in neuronal TNTs through a correlative cryo-electron microscopy (EM) approach by Zurzolo’s group (Sartori-Rupp et al., 2019).

Despite the conclusions proposed by Minhyeok Chan and colleagues about TNT formation being extremely intriguing, it is important to note that HEK and HeLa cells were used to produce wet data and conclusions cannot be automatically extended to CNS cells.

Several molecular players have been shown to positively or negatively regulate TNT formation, as reviewed by Ranabir Chakraborty et al. (Chakraborty et al., 2023a). However, the exact molecular pathways involved in TNT formation between neural cells remain largely unclear (as detailed only for astrocytes in Table 1). As a result, there are currently no molecular targets that can be used to design TNT-specific interfering experiments aimed at selectively destroying TNTs while preserving other cell protrusions. This greatly hinders the identification of TNT’s functions.

The only way to interfere with TNT formation currently is through the use of F-actin depolymerizing drugs such as Cytochalasin-D, Latrunculin B and Jasplakinolide. However, this treatment affects other F-actin dependent mechanisms in addition to TNT formation. Another possible approach is the use of not-in-touch coculture, which involves the use of the Transwell® system. Depending on the size-porosity of the separating membrane, not only TNTs but also other long-range intercellular trafficking mechanisms can be prevented. However, the literature is controversial and not aligned on the size-porosity required to exclusively exclude TNT-mediated intercellular trafficking.

Similarly, there is currently no possibility to specifically stimulate TNT formation. Cell stress conditions such as exposure to UV radiation (Hajek et al., 1989), oxidative stress (Wang et al., 2011), hypoxia (Desir et al., 2016; Yang et al., 2020), and other cell stressors upregulate TNT formation, but, under these conditions, mechanisms other than the formation of TNT are also induced.

In summary, the lack of specific knowledge about the molecules and pathways involved in TNT formation in neural cells, as well as the absence of TNT-specific triggering and destroying factors, are areas that need improvement to design more specific experiments aimed at isolating the specific contribute of TNTs useful at identifying TNT’s functions.

## 4 Dynamic and structural organization of TNTs in CNS-cells: from 2D-models to *in-vivo* brain imaging

### 4.1 Microscopic challenges emerge

TNT-like connections have been found in human fetal brain sections between pericytes and between pericytes and endothelial cells, suggesting that these elements may play a role in the initial stages of brain vascularization (Errede et al., 2018). Fixed and live-cell experiments have shown that astrocytes, oligodendrocytes, and neurons can all form TNTs in both physiological and pathological conditions (as reviewed by Khattar and colleagues (Khattar et al., 2022)). Additionally, in recent research, we have shown that TNTs occur between human blood-brain barrier cells (Pisani et al., 2022). While these studies have demonstrated different TNT-mediated transport of cellular cargoes and stimuli in both physiological and pathological 2D *in vitro* models, conclusions regarding TNT functions remain confined to the *in vitro* model used, and whether TNTs exist in multicellular 3D-CNS models and in CNS in general remains unclear.

However, the use of 3D-CNS models such as human mini-brains and murine models generated through induced human pluripotent stem cell (iPSC) technology and cell-specific differentiation could strongly contribute to filling this gap. These models recapitulate *in vivo* tissue architecture more effectively than neural 2D-cultures [as reviewed in (Hou and Kuo, 2022; Kofman et al., 2022; Wang et al., 2023)] and could help investigate TNT functions in CNS physiology (as detailed in Table 1). Although the use of 3D models is largely accessible for many laboratories, whether TNTs exist in these models remains unknown.

A recent technical advance in microscopy could revolutionize TNT analysis in the 3D models and in CNS slices. Zurzolo’s group recently analyzed the transient external granular layer of the developing cerebellum through high-resolution, serial-sectioning, scanning electron microscopy supported by 3D-reconstruction and deep-learning approaches. This innovative approach has revealed unprecedented details about the spatial-temporal connectivity between neural cells (Cordero Cervantes et al., 2023) and could represent a new frontier for TNT identification in CNS sections. More specifically, this study highlights the potential of a newly developed software designed for tracing cell-to-cell connections in 3D samples. The software utilized for this purpose is CellWalker, which is accessible at (https://github.com/utraf-pasteur-institute/CellWalker). CellWalker streamlines the processing of segmented microscopy images, thereby simplifying the identification of intercellular bridges within 3D images. This could significantly aid in the identification of TNT-like structures in CNS sections.

The step forward towards demonstrating the existence of TNTs in the living CNS was published by Alarcon-Martinez and colleagues in two recent, elegant *in vivo* studies. For the first time, the authors showed that TNTs exist in the mouse retina. Specifically, they demonstrated that inter-pericyte TNTs-mediated (IP-TNTs) intercellular Ca2+ waves control neurovascular coupling in the retina in physiological conditions, and that this mechanism is altered in glaucoma (Alarcon-Martinez et al., 2020; Alarcon-Martinez et al., 2022).

In these papers, non-invasive live retinal imaging using two-photon laser-scanning microscopy was used to investigate IP-TNTs function *in vivo*. The authors found that IP-TNTs had an open-ended proximal side and a closed-ended terminal (end-foot) that joined with distal pericyte processes via gap junctions. They also discovered that IP-TNTs transport organelles such as mitochondria, which can move along these processes, and act as a conduit for intercellular Ca2+ waves, mediating communication between pericytes. These data represent the first and only available data about TNTs in the living retina.

However, the question remains: do TNTs also exist in the living brain? To investigate this possibility, specific tools and animal models are required. One useful tool is based on the expression of cell-type-specific fluorescent proteins, ion trackers, and organelle-specific fluorescent proteins in a CNS-cell type-specific manner. For example, mice that express red fluorescent protein (Ds-RED) under the control of the NG2 promoter (Cspg4) can help identify NG2-positive pericytes in the central nervous system. Another tool is the expression of the genetically encoded Ca2+ indicator GCaMP6 under the NG2 promoter, which enables the tracing of calcium dynamics in NG2-positive pericytes. Animal models that express a mitochondrial-specific version of Dendra2 in a cell-specific manner can also be used. These tools are commercially available from The Jackson Laboratory and were used in previous studies (Alarcon-Martinez et al., 2020; Alarcon-Martinez et al., 2022). However, it should be noted that these animal models do not allow for the unequivocal distinction of cell types expressing the proteins of interest. Furthermore, it would be useful to have various fluorescent cargoes available, in order to discriminate cargo-specific differences in the TNTs-mediated intercellular transfer. This represents a significant obstacle for the *in vivo* study of TNTs.

What is the main limiting factor for investigating TNTs using *in vivo* approaches when animal models are available? The real limit is represented by the microscopic approaches needed to correctly describe the dynamics and structure of TNTs in the living CNS, especially in the living brain. The reason for this technical limit is intrinsic to the structure and dynamics of TNTs, which pose different microscopic challenges for visualizing their dynamics at a nanometric resolution in the brain.

TNTs are thin (20–700 nm) and highly dynamic structures. In just a few minutes, they can assemble, transport an organelle (hundreds of nanometers), or a molecule (sub-nanometers), and transfer these objects to another distant cell. How can we describe this rapid process with high spatial-temporal resolution while preserving cells from phototoxicity? Notably, phototoxicity is particularly crucial for TNT analysis, as it could induce TNT formation. This represents the main point that must be addressed in live-cell microscopy approaches useful for investigating TNTs in 2D and 3D models and *in vivo*.

The major approaches used in the literature for live-cell TNT analysis in 2D models are based on spinning-disk microscopy (SDM) approaches. This approach is widely used to describe neural complexity and functionality while preserving neurons from phototoxicity (Manzella-Lapeira et al., 2021).

Another opportunity comes from the use of holotomography (3D holographic) microscopy (HM), which is a label-free live-cell imaging approach recently reported for TNT investigation by Hans Zoellner and colleagues (Zoellner et al., 2020).

More recently, three-dimensional multi-color live-cell super-resolution imaging at high speed was achieved through the Structured Illumination Microscopy (SIM) approach. In SIM, the sample is illuminated with patterned light to minimize photon dose. After image acquisition, dedicated software analyzes the information, obtaining a reconstruction with a resolution about 2-fold higher than the diffraction limit. The spatial resolution of commercially available SIM microscopes is similar to other super-resolution microscopy techniques (e.g., STED and STORM), but the temporal resolution of SIM is better. In addition, SIM requires lower light intensity for imaging compared to STED and STORM, strongly preserving cell integrity and reducing phototoxicity more that SDM (reviewed in (Badawi and Nishimune, 2020). Furthermore, SIM can also be used for thicker sections, such as organotypic brain slice cultures (Olenick et al., 1988) and, recently, was further improved using a rationalized deep learning approach pushing the super-resolution limit of the technique in live-imaging of subcellular processes (Qiao et al., 2023). Consequently, SIM appears to be more appropriate for investigating the high dynamicity of TNTs at a nanometric resolution. Considering the various microscopy approaches currently employed, it is evident that much remains to be done to achieve an effective analysis of TNTs *in vivo*.

## 5 Conclusion

Upon reviewing the current literature on TNTs-based intercellular crosstalk, it seems that AI approaches, 3D *in-vitro* and animal models, and microscopic techniques currently available, could be useful in deciphering TNT’s structure, functions, and dynamics (Figure 1). In particular, super-resolution microscopy such as SIM, which is useful for analyzing live cells at nanometric resolution, could represent a great opportunity towards this aim.

Given the range of possibilities currently available, we believe that the question of whether TNTs are present in the brain could be successfully addressed in the near future. This breakthrough could represent a new frontier in neuroscience, providing valuable insight into intercellular connectivity in the brain.

## References

[B1] Abounit S. Bousset L. Loria F. Zhu S. de Chaumont F. Pieri L. (2016). Tunneling nanotubes spread fibrillar α-synuclein by intercellular trafficking of lysosomes. EMBO J. 35 (19), 2120–2138. 10.15252/embj.201593411 27550960 PMC5048354

[B2] Alarcon-Martinez L. Shiga Y. Villafranca-Baughman D. Belforte N. Quintero H. Dotigny F. (2022). Pericyte dysfunction and loss of interpericyte tunneling nanotubes promote neurovascular deficits in glaucoma. Proc. Natl. Acad. Sci. U. S. A. 119 (7), e2110329119. 10.1073/pnas.2110329119 35135877 PMC8851476

[B3] Alarcon-Martinez L. Villafranca-Baughman D. Quintero H. Kacerovsky J. B. Dotigny F. Murai K. K. (2020). Interpericyte tunnelling nanotubes regulate neurovascular coupling. Nature 585 (7823), 91–95. 10.1038/s41586-020-2589-x 32788726

[B4] Badawi Y. Nishimune H. (2020). Super-resolution microscopy for analyzing neuromuscular junctions and synapses. Neurosci. Lett. 715, 134644. 10.1016/j.neulet.2019.134644 31765730 PMC6937598

[B5] Ceran Y. Ergüder H. Ladner K. Korenfeld S. Deniz K. Padmanabhan S. (2022). TNTdetect.AI: A deep learning model for automated detection and counting of tunneling nanotubes in microscopy images. Cancers 14 (19), 4958. 10.3390/cancers14194958 36230881 PMC9562025

[B6] Chakraborty R. Belian S. Zurzolo C. (2023a). Hijacking intercellular trafficking for the spread of protein aggregates in neurodegenerative diseases: A focus on tunneling nanotubes (TNTs). Extracell. Vesicles Circ. Nucleic Acids 4, 27–43. 10.20517/evcna.2023.05 PMC11648486 39698299

[B7] Chakraborty R. Nonaka T. Hasegawa M. Zurzolo C. (2023b). Tunnelling nanotubes between neuronal and microglial cells allow bi-directional transfer of α-Synuclein and mitochondria. Cell. Death Dis. 14 (5), 329–412. 10.1038/s41419-023-05835-8 37202391 PMC10195781

[B8] Chang M. Lee O. C. Bu G. Oh J. Yunn N. O. Ryu S. H. (2022). Formation of cellular close-ended tunneling nanotubes through mechanical deformation. Sci. Adv. 8 (13), eabj3995. 10.1126/sciadv.abj3995 35353579 PMC8967236

[B9] Chen J. Cao J. (2021). Astrocyte-to-neuron transportation of enhanced green fluorescent protein in cerebral cortex requires F-actin dependent tunneling nanotubes. Sci. Rep. 11 (1), 16798. 10.1038/s41598-021-96332-5 34408233 PMC8373867

[B10] Cordero Cervantes D. Khare H. Wilson A. M. Mendoza N. D. Coulon-Mahdi O. Lichtman J. W. (2023). 3D reconstruction of the cerebellar germinal layer reveals tunneling connections between developing granule cells. Sci. Adv. 9 (14), eadf3471. 10.1126/sciadv.adf3471 37018410 PMC10075961

[B11] Cordero Cervantes D. Zurzolo C. (2021). Peering into tunneling nanotubes-The path forward. EMBO J. 40 (8), e105789. 10.15252/embj.2020105789 33646572 PMC8047439

[B12] Desir S. Dickson E. L. Vogel R. I. Thayanithy V. Wong P. Teoh D. (2016). Tunneling nanotube formation is stimulated by hypoxia in ovarian cancer cells. Oncotarget 7 (28), 43150–43161. 10.18632/oncotarget.9504 27223082 PMC5190014

[B13] Dilna A. Deepak K. V. Damodaran N. Kielkopf C. S. Kagedal K. Ollinger K. (2021). Amyloid-β induced membrane damage instigates tunneling nanotube-like conduits by p21-activated kinase dependent actin remodulation. Biochim. Biophys. Acta Mol. Basis Dis. 1867 (12), 166246. 10.1016/j.bbadis.2021.166246 34403739

[B14] Errede M. Mangieri D. Longo G. Girolamo F. de Trizio I. Vimercati A. (2018). Tunneling nanotubes evoke pericyte/endothelial communication during normal and tumoral angiogenesis. Fluids Barriers CNS 15 (1), 28. 10.1186/s12987-018-0114-5 30290761 PMC6173884

[B15] Girolamo F. Lim Y. P. Virgintino D. Stonestreet B. S. Chen X. F. (2023). Inter-Alpha inhibitor proteins modify the microvasculature after exposure to hypoxia-ischemia and hypoxia in neonatal rats. Int. J. Mol. Sci. 24 (7), 6743. 10.3390/ijms24076743 37047713 PMC10094872

[B16] Hajek M. Meier-Ewert K. Wirz-Justice A. Tobler I. Arendt J. Dick H. (1989). Bright white light does not improve narcoleptic symptoms. Eur. Arch. Psychiatry Neurol. Sci. 238 (4), 203–207. 10.1007/BF00381466 2759154

[B17] Hodneland E. Lundervold A. Gurke S. Tai X. C. Rustom A. Gerdes H. H. (2006). Automated detection of tunneling nanotubes in 3D images. Cytom. Part J. Int. Soc. Anal. Cytol. 69 (9), 961–972. 10.1002/cyto.a.20302 16969816

[B18] Hou P. S. Kuo H. C. (2022). Central nervous system organoids for modeling neurodegenerative diseases. IUBMB Life 74 (8), 812–825. 10.1002/iub.2595 35102668

[B19] Jung E. Osswald M. Blaes J. Wiestler B. Sahm F. Schmenger T. (2017). Tweety-homolog 1 drives brain colonization of gliomas. J. Neurosci. Off. J. Soc. Neurosci. 37 (29), 6837–6850. 10.1523/JNEUROSCI.3532-16.2017 PMC6705725 28607172

[B20] Khattar K. E. Safi J. Rodriguez A. M. Vignais M. L. (2022). Intercellular communication in the brain through tunneling nanotubes. Cancers 14 (5), 1207. 10.3390/cancers14051207 35267518 PMC8909287

[B21] Kofman S. Mohan N. Sun X. Ibric L. Piermarini E. Qiang L. (2022). Human mini brains and spinal cords in a dish: modeling strategies, current challenges, and prospective advances. J. Tissue Eng. 13, 204173142211133. 10.1177/20417314221113391 PMC9310295 35898331

[B22] Li A. Han X. Deng L. Wang X. (2022). Mechanical properties of tunneling nanotube and its mechanical stability in human embryonic kidney cells. Front. Cell. Dev. Biol. 10, 955676. 10.3389/fcell.2022.955676 36238686 PMC9551289

[B23] Ljubojevic N. Henderson J. M. Zurzolo C. (2021). The ways of actin: why tunneling nanotubes are unique cell protrusions. Trends Cell. Biol. 31 (2), 130–142. 10.1016/j.tcb.2020.11.008 33309107

[B24] Manzella-Lapeira J. Brzostowski J. Serra-Vinardell J. (2021). Studying neuronal biology using spinning disc confocal microscopy. Methods Mol. Biol. Clifton N. J. 2304, 265–283. 10.1007/978-1-0716-1402-0_14 34028722

[B25] Olenick J. G. Wolff R. Nauman R. K. McLaughlin J. (1988). A flagellar pocket membrane fraction from trypanosoma brucei rhodesiense: immunogold localization and nonvariant immunoprotection. Infect. Immun. 56 (1), 92–98. 10.1128/IAI.56.1.92-98.1988 3335412 PMC259240

[B26] Pisani F. Castagnola V. Simone L. Loiacono F. Svelto M. Benfenati F. (2022). Role of pericytes in blood-brain barrier preservation during ischemia through tunneling nanotubes. Cell. Death Dis. 13 (7), 582. 10.1038/s41419-022-05025-y 35790716 PMC9256725

[B27] Qiao C. Li D. Liu Y. Zhang S. Liu K. Liu C. (2023). Rationalized deep learning super-resolution microscopy for sustained live imaging of rapid subcellular processes. Nat. Biotechnol. 41 (3), 367–377. 10.1038/s41587-022-01471-3 36203012

[B28] Rostami J. Holmqvist S. Lindström V. Sigvardson J. Westermark G. T. Ingelsson M. (2017). Human astrocytes transfer aggregated alpha-synuclein via tunneling nanotubes. J. Neurosci. Off. J. Soc. Neurosci. 37 (49), 11835–11853. 10.1523/JNEUROSCI.0983-17.2017 PMC5719970 29089438

[B29] Rustom A. Saffrich R. Markovic I. Walther P. Gerdes H. H. (2004). Nanotubular highways for intercellular organelle transport. Science 303 (5660), 1007–1010. 10.1126/science.1093133 14963329

[B30] Sartori-Rupp A. Cordero Cervantes D. Pepe A. Gousset K. Delage E. Corroyer-Dulmont S. (2019). Correlative cryo-electron microscopy reveals the structure of TNTs in neuronal cells. Nat. Commun. 10 (1), 342. 10.1038/s41467-018-08178-7 30664666 PMC6341166

[B31] Scheiblich H. Dansokho C. Mercan D. Schmidt S. V. Bousset L. Wischhof L. (2021). Microglia jointly degrade fibrillar alpha-synuclein cargo by distribution through tunneling nanotubes. Cell. 184 (20), 5089–5106.e21. 10.1016/j.cell.2021.09.007 34555357 PMC8527836

[B32] Smirnov M. S. Garrett T. R. Yasuda R. (2018). An open-source tool for analysis and automatic identification of dendritic spines using machine learning. PloS One 13 (7), e0199589. 10.1371/journal.pone.0199589 29975722 PMC6033424

[B33] Sun X. Wang Y. Zhang J. Tu J. Wang X. J. Su X. D. (2012). Tunneling-nanotube direction determination in neurons and astrocytes. Cell. Death Dis. 3 (12), e438. 10.1038/cddis.2012.177 23222508 PMC3542613

[B34] Tardivel M. Bégard S. Bousset L. Dujardin S. Coens A. Melki R. (2016). Tunneling nanotube (TNT)-mediated neuron-to neuron transfer of pathological Tau protein assemblies. Acta Neuropathol. Commun. 4 (1), 117. 10.1186/s40478-016-0386-4 27809932 PMC5096005

[B35] Wang L. Owusu-Hammond C. Sievert D. Gleeson J. G. (2023). Stem cell-based organoid models of neurodevelopmental disorders. Biol. Psychiatry 93 (7), 622–631. 10.1016/j.biopsych.2023.01.012 36759260 PMC10022535

[B36] Wang X. Bukoreshtliev N. V. Gerdes H. H. (2012). Developing neurons form transient nanotubes facilitating electrical coupling and calcium signaling with distant astrocytes. PloS One 7 (10), e47429. 10.1371/journal.pone.0047429 23071805 PMC3469499

[B37] Wang X. Gerdes H. H. (2012). Long-distance electrical coupling via tunneling nanotubes. Biochim. Biophys. Acta 1818 (8), 2082–2086. 10.1016/j.bbamem.2011.09.002 21930113

[B38] Wang X. Veruki M. L. Bukoreshtliev N. V. Hartveit E. Gerdes H. H. (2010). Animal cells connected by nanotubes can be electrically coupled through interposed gap-junction channels. Proc. Natl. Acad. Sci. U. S. A. 107 (40), 17194–17199. 10.1073/pnas.1006785107 20855598 PMC2951457

[B39] Wang Y. Cui J. Sun X. Zhang Y. (2011). Tunneling-nanotube development in astrocytes depends on p53 activation. Cell. Death Differ. 18 (4), 732–742. 10.1038/cdd.2010.147 21113142 PMC3131904

[B40] Yang Y. Ye G. Zhang Y. L. He H. W. Yu B. Q. Hong Y. M. (2020). Transfer of mitochondria from mesenchymal stem cells derived from induced pluripotent stem cells attenuates hypoxia-ischemia-induced mitochondrial dysfunction in PC12 cells. Neural Regen. Res. 15 (3), 464–472. 10.4103/1673-5374.266058 31571658 PMC6921344

[B41] Zoellner H. Paknejad N. Cornwell J. A. Chami B. Romin Y. Boyko V. (2020). Potential hydrodynamic cytoplasmic transfer between mammalian cells: cell-projection pumping. Biophys. J. 118 (6), 1795–1860. 10.1016/j.bpj.2020.03.013 32234501 PMC7136330

[B42] Zurzolo C. (2021). Tunneling nanotubes: reshaping connectivity. Curr. Opin. Cell. Biol. 71, 139–147. 10.1016/j.ceb.2021.03.003 33866130

